# P-965. Effectiveness of Augmented Prospective Audit and Feedback in Reducing Carbapenem Use Following Discontinuation of Preauthorization

**DOI:** 10.1093/ofid/ofaf695.1165

**Published:** 2026-01-11

**Authors:** Yeonjin Son, Wongu Kang, Jiae Kim, Kyungkeun Cho, Inah Park, So Yun Lim, Seongman Bae, Sung-Han Kim

**Affiliations:** Asan Medical Center, Seoul, Republic of Korea, Seoul, Seoul-t'ukpyolsi, Republic of Korea; Asan Medical Center, Seoul, Republic of Korea, Seoul, Seoul-t'ukpyolsi, Republic of Korea; Asan Medical Center, Seoul, Republic of Korea, Seoul, Seoul-t'ukpyolsi, Republic of Korea; Asan Medical Center, Seoul, Republic of Korea, Seoul, Seoul-t'ukpyolsi, Republic of Korea; Asan Medical Center, Seoul, Republic of Korea, Seoul, Seoul-t'ukpyolsi, Republic of Korea; National Medical Center , Seoul, Seoul-t'ukpyolsi, Republic of Korea; Asan Meidical Center, Songpa-gu, Seoul-t'ukpyolsi, Republic of Korea; Asan medical center, Seoul, Seoul-t'ukpyolsi, Republic of Korea

## Abstract

**Background:**

At a tertiary hospital in Korea, antimicrobial stewardship program (ASP) activities, including preauthorization for carbapenem use, were suspended in early 2024 due to a nationwide medical strike, leading to increased carbapenem consumption. In response, a PAF strategy was introduced in December 2024 and further enhanced in January 2025 to include compliance monitoring, ward rounds, and follow-up feedback. This study evaluated the impact of the augmented PAF on carbapenem use following ASP suspension and assessed changes in prescriber adherence compared to standard PAF.

**Figure:**
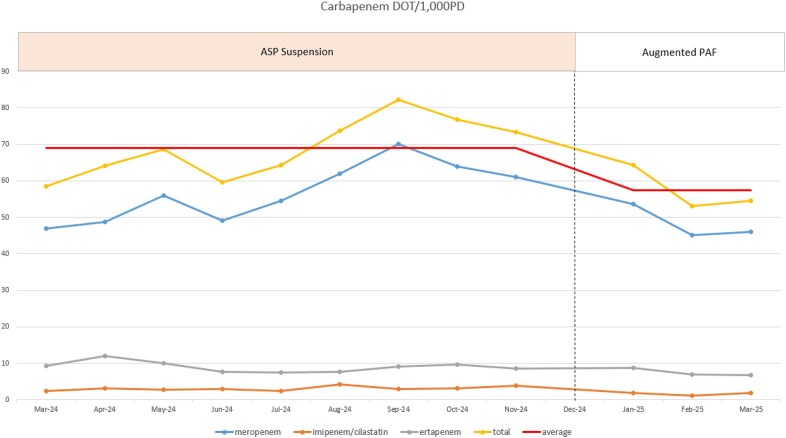
Trend in carbapenem use expressed as monthly DOT per 1,000 patient-days Carbapenem DOT/1,000PD decreased after implementation of the augmented prospective audit and feedback (PAF) strategy. December 2024 (standard PAF only) was excluded as transition period.

**Figure:**
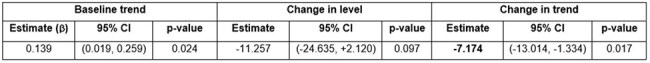
Estimated Changes in Carbapenem Use Following ASP Suspension and Augmented PAF Implementation Interrupted time series analysis showing a significant reduction in DOT during augmented PAF period compared to ASP suspension period (p=0.017)

**Methods:**

We retrospectively analyzed monthly carbapenem Days of Therapy (DOT) from January 2021 to March 2025. DOT trends were assessed using interrupted time series analyses based on a linear regression model, comparing the ASP suspension period (March–November 2024) with the subsequent period following implementation of the augmented PAF (January–March 2025). A separate comparison of prescriber adherence rates was conducted between December 2024 (standard PAF only) and January–March 2025 (augmented PAF).

**Figure:**
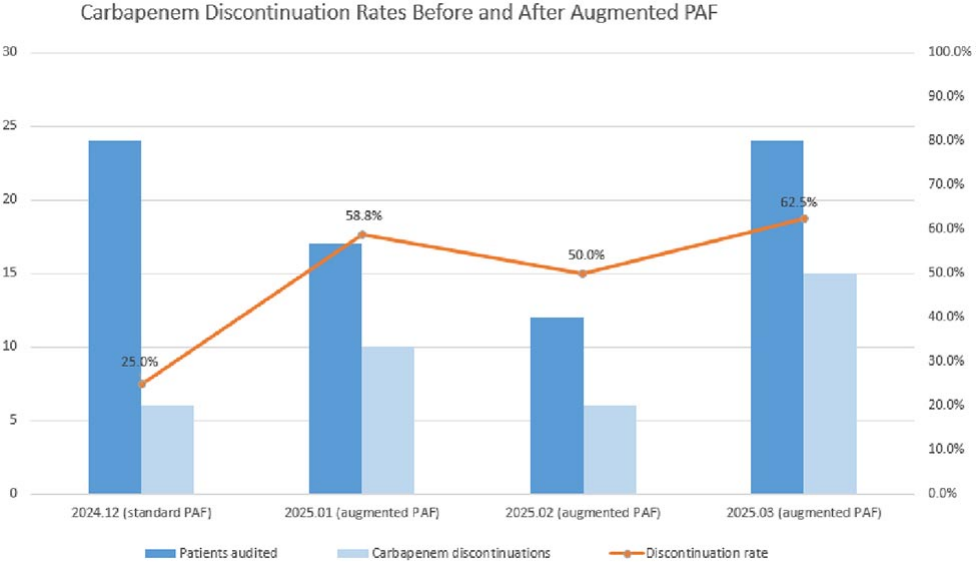
Number of Patients audited, Carbapenem Discontinuations, and Discontinuation Rates Before and After Augmented PAF Implementation

In December 2024 (standard PAF), 7-day natural discontinuations were assessed, whereas from January to March 2025, post-intervention discontinuations were analyzed. Augmented PAF implementation was associated with an increased rate of carbapenem discontinuation.

**Results:**

Following the introduction of the augmented PAF in January 2025, a significant reversal in carbapenem DOT trend was observed, marked by a monthly decrease of 7.174 DOT (95% CI: –13.014 to –1.334; p = 0.017) and a relative reduction of 30.9%. In December 2024, when only standard PAF was implemented, 25.0% (6/24) of patients noncompliant with initial feedback discontinued or modified carbapenem use within 7 days, whereas this rate increased to 58.5% (31/53) following the introduction of augmented PAF with additional feedback after clinical ward rounds from January to March 2025 (p = 0.008).

**Conclusion:**

PAF remains an important core activity of antimicrobial stewardship alongside preauthorization. In this study, implementation of PAF was associated with a reduction in carbapenem use following ASP suspension, and the adoption of an augmented PAF approach incorporating clinical rounding and real-time feedback improved prescriber adherence.

**Disclosures:**

All Authors: No reported disclosures

